# Highly-efficient (>70%) and Wide-spectral (400–1700 nm) sub-micron-thick InGaAs photodiodes for future high-resolution image sensors

**DOI:** 10.1038/s41377-024-01652-6

**Published:** 2024-11-15

**Authors:** Dae-Myeong Geum, Jinha Lim, Junho Jang, Seungyeop Ahn, SeongKwang Kim, Joonsup Shim, Bong Ho Kim, Juhyuk Park, Woo Jin Baek, Jaeyong Jeong, SangHyeon Kim

**Affiliations:** 1 School of Electrical Engineering, Korea Advanced Institute of Science and Technology (KAIST), Daejeon, 34141 Republic of Korea; 2 Department of Electrical and Computer Engineering, Inha University, Incheon, 22212 Republic of Korea

**Keywords:** Optoelectronic devices and components, Imaging and sensing

## Abstract

This paper demonstrates the novel approach of sub-micron-thick InGaAs broadband photodetectors (PDs) designed for high-resolution imaging from the visible to short-wavelength infrared (SWIR) spectrum. Conventional approaches encounter challenges such as low resolution and crosstalk issues caused by a thick absorption layer (AL). Therefore, we propose a guided-mode resonance (GMR) structure to enhance the quantum efficiency (QE) of the InGaAs PDs in the SWIR region with only sub-micron-thick AL. The TiO_x_/Au-based GMR structure compensates for the reduced AL thickness, achieving a remarkably high QE (>70%) from 400 to 1700 nm with only a 0.98 μm AL InGaAs PD (defined as 1 μm AL PD). This represents a reduction in thickness by at least 2.5 times compared to previous results while maintaining a high QE. Furthermore, the rapid transit time is highly expected to result in decreased electrical crosstalk. The effectiveness of the GMR structure is evident in its ability to sustain QE even with a reduced AL thickness, simultaneously enhancing the transit time. This breakthrough offers a viable solution for high-resolution and low-noise broadband image sensors.

## Abstract (web summary)

Sub-micron-thick InGaAs PD using guided-mode resonance for advanced high-resolution SWIR image sensors for wide band, high efficiency, and low X-talk

## Graphical abstract

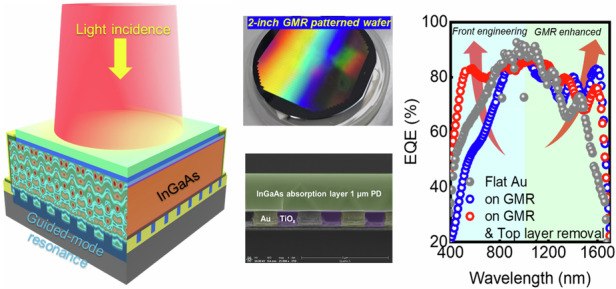

## Introduction

Short-wavelength infrared (SWIR) imaging has significantly contributed to technological advances and has found diverse applications, including night-vision systems and the structural clarification of unknown substances in various fields such as industrial, science, and security^1–3^. The importance of detecting light beyond the visible spectrum has grown, leading to an increased demand for the development of SWIR image sensors to provide more valuable and accurate information. Therefore, ultra-broadband detection beyond a single spectrum range is crucial for enabling small form factors and multicolor imaging within a limited device area^4^. Broadband image sensors allow the various subpixels to be composed of R, G, B, and SWIR in a single pixel with color filters, which can provide high-speed imaging and overcoming the image fusion techniques obtained by different imaging devices. To meet these demands, substantial efforts have been invested for broadband detectors by employing various 2D/3D materials, optical design, and integration techniques^4–9^.

Among them, one of the main pathways is the use of silicon (Si)-based image sensor platforms, highlighting CMOS compatibility and high resolution. Colloidal quantum dots have been utilized to extend the detection range to the SWIR region, demonstrating high resolution and broadband performance, albeit with slightly lower external quantum efficiencies (EQEs) and inherently weak reliability^10^. In contrast, III-V compound semiconductors, such as InGaAs, have been widely used due to their superior absorption in the SWIR region and high carrier mobility. By utilizing the InGaAs with high QE in the SWIR region, many researchers have attempted to cover the visible spectrum region including the vertical integration, and the InP substrate thinning^11–13^. Typically, the InP substrate thinning approach has been utilized for enhancing visible light absorption and maintaining QE for SWIR region in conventional flip-chip bonding structure^12,13^. They showed the ability to cover the broadband spectrum from visible to SWIR region, although resolution was relatively lower compared to CMOS image sensors (CIS). Recently, various approaches have been investigated for better resolution in InGaAs broadband image sensors by improving integration methodologies such as Cu-Cu bump bonding, and monolithic 3D (M3D) integration with lithographic alignment, low thermal budget, and CMOS-like process^12–15^.

However, despite the various integration methods developed to obtain high-resolution and broadband image sensors, fundamental limitations of the pixel itself remain a significant challenge. One of the key issues is the trade-off between the external quantum efficiency (EQE) and the absorption layer thickness. To maximize QE in the SWIR region, InGaAs photodiode (PD) pixels require a very thick absorption layer (AL) above 3 μm, which induces several problems. First, the thick AL complicates the lithography process due to the mismatch in focusing depth across the samples in the lithography tool. Additionally, for high-resolution imaging requiring mega or gigapixels, the slow response speed of pixels hinders obtaining a high frame rate in imaging^16,17^. A thick AL limits the response speed in the slow transit time limited operation region, making a fast transit time desirable for high-frame-rate imaging. Furthermore, optical and electrical crosstalk issues arise due to the thick AL in InGaAs PDs, which have long diffusion lengths and no isolation process, leading to increased collection time for photogenerated carrier separation and reduced EQE due to recombination of the photo-generated carriers^18–21^.

Considering these issues, it is clear that future broadband/high-resolution image sensors require a thin AL structure capable of applying to the M3D fabrication process. They must maintain broadband absorption performances with high QE across the entire spectrum region while improving response speed to achieve fast transit times for high-frame-rate imaging and low optical/electrical crosstalk. Current InGaAs SWIR image sensors achieve broadband absorption by a thick AL above 3 μm and removing the substrate, but they do not address other problems related to the fundamental trade-off between AL and other performance parameters. Therefore, designing ultra-thin but high QE absorber structures is inevitable for future InGaAs broadband/high-resolution image sensors.

In this paper, we first present sub-micron-thick InGaAs broadband photodetectors (PDs) with a high QE exceeding 70% across a range from 400 nm to 1700 nm by introducing an optical confinement structure and newly designing the epitaxial layers to maximize optical absorption with an extremely thin AL. To fully leverage the advantages of InGaAs, including broadband absorption, a trade-off between each layer’s thickness and QE should be considered through simulating the absorption behavior. These PDs were designed to be compatible with traditional flip-chip bonding and M3D integration (Sequential fabrication), aiming to achieve high resolution imagers. Additionally, to meet the requirements of broadband performances and rapid transit time, we decreased the AL thickness (*T*_AL_) by incorporating a thin surface doping layer and introducing a TiO_x_/Au-based guided-mode resonance (GMR) structure. This alters the double-pass absorption to multi-resonant absorption, with a strong resonance occurring in the thin InGaAs AL, reduced to 750 nm. As a result, we have achieved broadband InGaAs PDs with superior performance, achieving approximately 70% EQE across all wavelength bands from visible to SWIR.

## Results

### Design of front-epitaxial structure and rear-side GMR structure

**Fig. 1:**
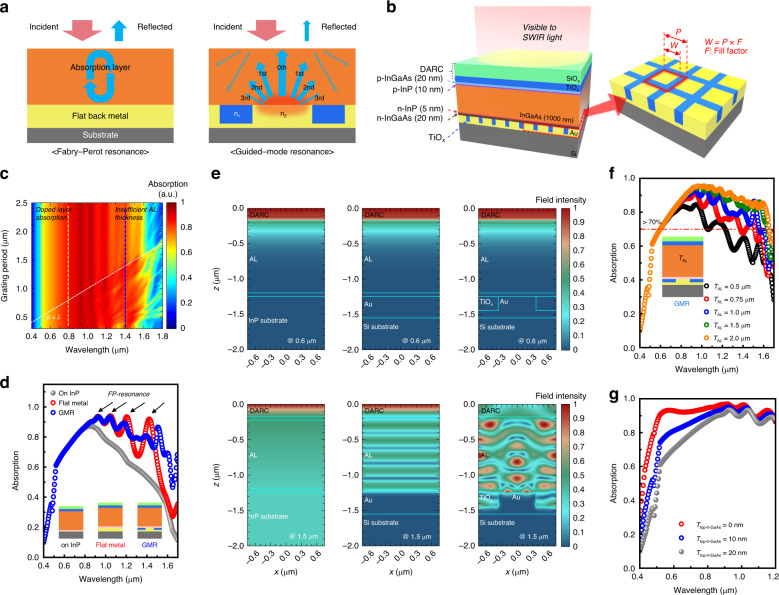
Scheme and principle of the proposed structure for light absorption in a broadband spectrum range. **a** Schematics of conventional Fabry-Perot resonance cavity and proposed GMR structure. **b** Design of GMR integrated InGaAs PD structures and design parameters of GMR structure. **c** 2D mapping results for the relative amount of absorption in AL grating period as a function of wavelength with fixed *T*_AL_ = 1 μm. **d** RCWA simulation results with 1 μm AL PD on rear side engineering about InP substrate, flat metal structure, and GMR structure. **e** Electric field intensity distribution for 1.0 μm AL InGaAs PIN PDs on different bottom structures at 0.6 μm and 1.5 μm. **f** *T*_AL_ dependent absorption spectra in terms of wavelength. **g** Top InGaAs layer thickness-dependent absorption spectra for visible light absorption

To reduce the *T*_AL_ in InGaAs-based SWIR PDs, compensation for reduced absorption at long wavelengths is crucial, especially when a small *T*_AL_ results in insufficient absorption at a long wavelength range. Figure 1a illustrates the proposed approach for compensating a small *T*_AL_ by extending an optical absorption path. Theoretically, reducing the *T*_AL_ by more than half might be challenging, as conventional In or Cu-based flip-chip bonding typically relies on Fabry-Perot resonance with a flat back metal, resulting in only double-pass light absorption^22,23^. Accordingly, a simple reduction in *T*_AL_ may not be a viable strategy within this conventional approach, highlighting the need for alternative methodologies to address the limitations. In this regard, we propose to introduce the guided-mode resonance (GMR) in the pixel for enhancing QE in SWIR region. It offers multiple resonant absorptions by engineering the optical structure for the GMR effect to satisfy optical resonances with specific diffraction orders at wavelengths. These further resonances with the diffracted light at different angles due to their effective refractive index at each wavelength can be achieved by varying the period, material, and fill factor (F.F) meaning the ratio of the metal area in a single period. Especially, the strong interference of the zeroth order resonant mode at certain wavelengths with the GMR structure leads to significant absorption under specific phase matching conditions. From this resonance phenomenon, both maintaining QE and reducing the *T*_AL_ in the InGaAs PD can be realized.

By employing periodic patterns under the AL region, the absorption at the long wavelength can be enhanced with strong multi-resonance in a thin *T*_AL_. To design the GMR structure, optical simulations using rigorous coupled-wave analysis (RCWA) were conducted using the epitaxial structure and design parameters displayed in Fig. 1b. The detailed epitaxial structures are shown in Fig. S1. Additionally, SiO_x_/TiO_x_ double-layer anti-reflection coating (DARC) layers are inserted on the top surface to minimize surface reflection loss of the incident light. Figure 1c illustrates the simulated spectra (400–1700 nm) of the relative absorption in the InGaAs AL as a function of the grating period for a 1-µm-thick InGaAs AL when F.F is 0.5. The detailed mode analysis was exhibited in Fig. S2. It is noted that similar absorption intensity was observed concerning the incident wavelength up to 1 μm for any grating periods. This indicates that absorption behavior in the shorter wavelength region, it is affected by the doped surface layer of InGaAs. Even the relatively thin 20-nm-thick InGaAs layer can reduce visible light absorption from 400 nm to 800 nm due to the free-carrier absorption. At the longer wavelength region, it is shown that the absorption performance was clearly enhanced when the period (*P*) was below the incident wavelength. In the GMR operation regime, the absorption due to the multiple resonances with the reflected beams of high order diffractions is dominant when *P* > *λ* condition. Meanwhile, the zeroth order diffraction becomes significant since the grating period is much smaller than the operating wavelength (*P* < *λ*). This strong GMR absorption provided a significant enhancement up to the 1.8 μm spectrum range even with a typically insufficient *T*_AL_ of 1 μm.

To quantitatively evaluate the absorption property for the GMR structure, we analyzed the simulation for the absorption spectra in the ALs of the three configurations such as the AL 1) on InP, 2) on flat metal on Si, and 3) on GMR structure (TiO_x_/Au) on Si, as shown in Fig. 1d. For reference, InGaAs PIN PD on the InP substrate (n-on-p) and InGaAs PD on the Si substrate (p-on-n) with a flat back metal structure were simultaneously simulated with the same epitaxial structure, as shown in Fig. 1b. It clearly shows that there is a large difference in broadband absorption property. In the visible light region from 400 nm to 800 nm, all devices show the same trend regardless of bottom structures. Beyond 800 nm, the amount of light absorption is dramatically reduced with InGaAs PD on the InP substrate due to insufficient *T*_AL_ and the dispersion of the extinction coefficient for the InGaAs^24^. Then, almost all of the light within the AL region passes through the whole layers toward the substrate without greater interfacial reflection effects.

In contrast to the AL on InP substrate, the periodical absorption resonant peaks which are induced by Fabry-Perot interferences in the spectrum indicate an enhanced optical absorption performance for the only flat back metal structure under AL. Supporting the FP resonances simultaneously, the GMR structure under the InGaAs AL can provide additional absorption based on the strong GMR effect above 1500 nm, although the flat metal structure allowed significant enhancement above 800 nm. From this result, it was noted that the GMR structure can allow improved broadband absorption property beyond the material’s cut-off band compared to the flat metal structure with the same *T*_AL_ by utilizing multiple resonances. Therefore, considering the photolithographic process capability and maximum absorption in the broadband region, the GMR dimensions were determined to consist of a TiO_x_ and Au width of 750 nm, a period of 1500 nm, and a height of 300 nm from the simulation. Furthermore, we evaluated the fabrication tolerance and engineering of resonance peak shift with the same structure as shown in Fig. S3 and Fig. S4. From these results, it was confirmed that the target wavelength could be tuned by adjusting bottom GMR variables with ±10% tolerance for GMR patterns

The resulting electric field distributions, depending on different bottom structures and wavelengths, were plotted for TE-polarized light with a 1 µm AL PD, as shown in Fig. 1e. Here, we selected two representative wavelengths of 600 nm and 1500 nm to evaluate the distinguishable resonant characteristics of the three structures, where all electric field distribution was normalized by the maximum field in each structure. The light of 600 nm wavelength could not induce resonance in all structures due to shorter wavelengths mainly being absorbed before reaching the back surface. It is consistent with the absorption behavior shown in Fig. 1d. On the other hand, at a 1500 nm wavelength, a different electric field distribution of the interference in each bottom structure was clearly observed. The constant electric field with a small intensity is distributed throughout the entire region in the InP bottom structure because of the very small indices contrast between every interface in the layer. Additionally, it is trivial that periodic interference patterns, induced by the Fabry–Perot cavity, are observed for the flat metal structure, while the stronger resonance induced by the bottom GMR structure occurs due to the constructive and destructive interferences with the multiple-diffracted beams in the AL. It suggests that the GMR structure could confine the electric field intensity well with multiple strong resonances at longer wavelengths despite the thin *T*_AL_.

Following the optimization of the bottom GMR structure for the given epitaxial layers, we investigated the *T*_AL_-dependent absorption behaviors with the fixed other layer thickness on the GMR structure, as shown in Fig. 1f. When the *T*_AL_ varied from 0.5 μm to 2.0 μm, the resulting spectra showed different FP resonance peaks depending on *T*_AL_ due to different cavity lengths. Below the wavelength of 700 nm, the absorption trend was fully consistent because the light absorption occurs through a single path interaction in the absorber regardless of the GMR structure at this wavelength range. On the other hand, the impact of the GMR structure was sufficiently apparent at longer wavelengths. With 1 μm and 0.75 μm of *T*_AL_, absorption slightly drops but still exhibits high absorption more than 70% in a range from 700 nm to 1700 nm. Simulations proved that sub-micron-thick PDs maintain more than 70% absorption of incident light at long wavelengths when employing the GMR structure, in contrast to the flat metal results, where a substantial loss in efficiency occurs above 1400 nm.

Lastly, to facilitate the broadband PDs from visible to SWIR, we additionally simulated the absorption enhancement by minimizing the optical loss at the front surface. In Fig. 1g, absorption spectra represent the clear enhancement depending on *T*_AL_ and top p-InGaAs thickness (*T*_top-InGaAs_) regarding the wavelength range from 0.4 μm to 1.2 μm. Here, it is noted that the *T*_top-InGaAs_ effect on absorption property in this experiment could lead to 1.2 times higher absorption efficiency just by tuning the *T*_top-InGaAs_ as shown in Fig. S5. Ultimately, based on these results, the optimal structure will be a thin p-InP layer and no p-InGaAs layer considering the visible light absorption. The optimum GMR structure design is determined for reducing the *T*_AL_ and *T*_top-InGaAs_ for high-resolution and ultra-broadband image sensors.

### Integration and fabrication process of GMR structure with InGaAs PDs

**Fig. 2:**
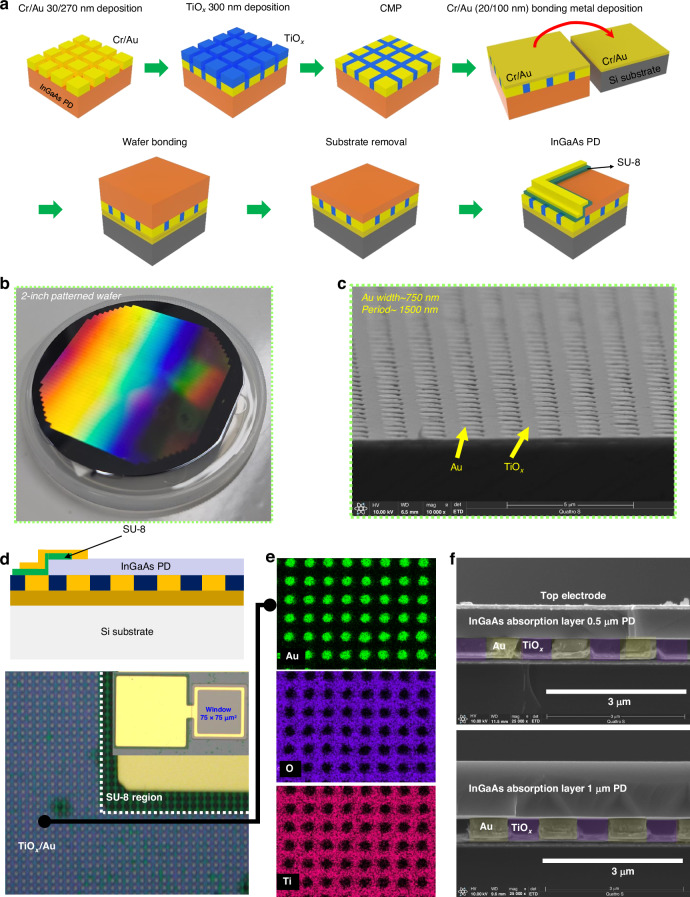
InGaAs SWIR pixel fabrication integrating with a GMR structure. **a** Schematics of the GMR integrated InGaAs PDs by utilizing wafer bonding based thin film transfer method **b** Photograph of wafer-level patterned GMR structure with 1.5 μm period. **c** SEM image for periodic patterns consisting of Au width of 0.75 μm and TiO_*x*_ 0.75 μm. **d** Schematic image of the fabricated PD on GMR structure and optical image of fabricated devices. **e** EDX images of Ti, O, and Au atoms at the top view. **f** Fabricated 0.5 μm and 1.0 μm AL InGaAs PD on GMR Si structure

Epitaxially grown InGaAs PIN PD structures were used by varying the *T*_AL_, as shown in Fig. S1. For comparison, a 2.1-μm-thick reference InGaAs PIN structure was used. Additionally, AL thinning structures have etch-stop layers consisting of InP/InGaAs layers for thin-film transfer on flat metal or GMR structures by wafer bonding^25–27^. The fabrication process for GMR integrated InGaAs PDs began with wafer-level patterning to form periodic structures using maskless lithography, as shown in Fig. 2a–c. The final devices were fabricated through isolation, SU-8 passivation, and the top metal deposition process. The detailed fabrication process is shown in the methods section. Devices were fabricated with different dimensions of 15 × 15, 30 × 30, 50 × 50, 75 × 75, and 100 × 100 μm² optical windows. Figure 2d shows the optical microscope (OM) image of the final device with the GMR structure. The inset figure represents the InGaAs PIN PD with a 75 × 75 μm² optical window. The TiO_x_/Au GMR structure was clearly observed underneath the InGaAs PDs in the magnified OM image. Furthermore, top-view energy-dispersive x-ray spectroscopy (EDX) images confirmed the formation of periodic TiO_x_ and Au, as shown in Fig. 2e. The cross-sectional SEM images of the 0.49 μm and 0.98 μm *T*_AL_ InGaAs PD (slight deviation from the target growth thickness, defined as 0.5 μm and 1 μm AL InGaAs PD) on Si with the GMR integration process are depicted in Fig. 2f. The device structures were successfully integrated on the TiO_x_/Au GMR structure on Si substrate, indicating that the integration and fabrication process are reliable and compatible with conventional hybrid integration, even in M3D integration. Also, the reference PD and integrated PD with flat metal were successfully fabricated (not shown here).

## Discussion

### Electrical and optical characterization of the fabricated PDs with different bottom structures

**Fig. 3:**
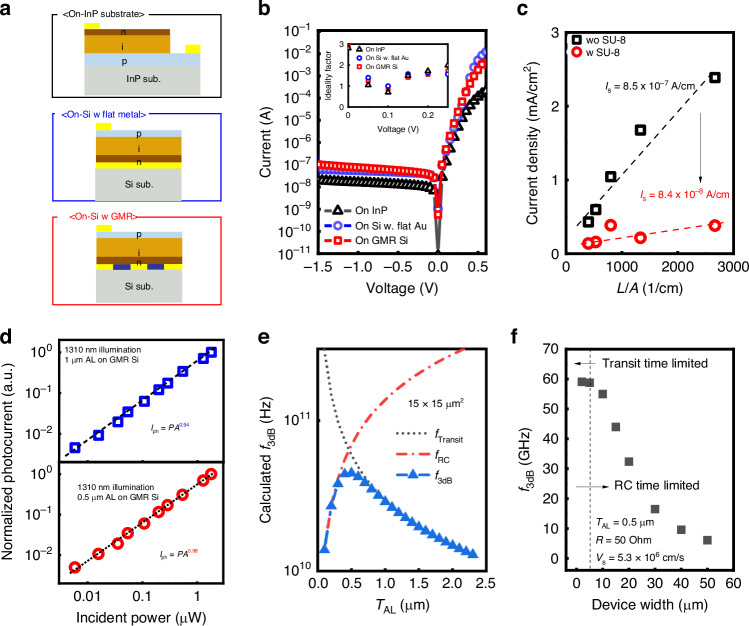
The performances of InGaAs PDs with three different bottom structures. **a** Schematics of the fabricated device structures with different bottom structures. **b** *I*–*V* characteristics for 1.0 μm AL InGaAs PD on InP, flat metal, GMR, and ideality factors as an inset figure (**c**) Surface leakage currents for 1 μm AL InGaAs PD with/without SU-8 passivation using size dependency. **d** *I*_ph_–*P*_in_ characteristics for 0.5 and 1 μm AL PDs on GMR Si. **e** Calculated *f*_3dB_ for 15 × 15 μm^2^ devices in terms of *T*_AL_. **f** Calculated *f*_3dB_ as a function of device width to confirm the transit time limited bandwidth

We investigated the electrical properties of the fabricated PDs, as shown in Fig. 3a. The schematics of the fabricated PDs depict the cross-sectional structures depending on the bottom structures. The reference samples on the InP substrate consist of an n-on-p structure, which was not inverted with the same epitaxial structure. As shown in Fig. 3b, all PDs with a 1 μm AL and a 100 μm × 100 μm optical window exhibited a good *I*_forward_/*I*_dark_ ratio of approximately >10^4^ at ±0.5 V. Transferred PDs show a slightly higher *I*_forward_, which could be attributed to the reduction in parasitic resistance due to the vertical PD structure. Furthermore, the ideality factors showed similar values in the low forward bias region, close to unity, indicating that the fabricated PDs maintained good crystal quality regardless of geometries and fabrication process.

Next, we evaluated the surface leakage current (*I*_s_) using size-dependent *I-V* measurements^28^. For obtaining high signal-to-noise (SNR) ratio, *I*_s_ should be suppressed for small pixels, which dominate the dark current. When the fabricated InGaAs PDs can sense actual blackbody radiation of 1000 K material emitting SWIR light, low dark current is a very important parameter, even with a high-quality absorber. Therefore, to suppress *I*_s_, SU-8 was used for surface passivation for InGaAs PDs, because SU-8 passivation has been studied for III–V compound semiconductors-based narrow bandgap PDs for suppressing *I*_s_^29,30^. The *I–V* data are shown in Fig. S6a–c. Figure S6c illustrates the extracted surface leakage current density by utilizing *J* = *J*_s_ × *L* + *J*_B_ × *A* resulting in values of 8.5 × 10^-7 ^A cm^-1^ and 8.4 × 10^-8 ^A cm^−1^ for without and with SU-8 passivation, where *J*, *J*_s_, *J*_B_, *L*, and *A* are current density, surface current density, bulk current density, perimeter, and area, respectively. To compare the leakage current, we analyzed the 500 nm *T*_AL_ devices as shown in Fig. S6d, e. It was observed that the dark current showed no noticeable degradation even with a *T*_AL_ of 500 nm, with or without passivation due to the InP/InGaAs heterojunction structure. Here, it was noted that the passivation effect of SU-8 is less than that of 1.0 μm AL PDs as shown in Fig. Sf. It is because the current would increase by diffusion current in the p-n junction, as the intrinsic layer thickness decreases. Therefore, for 0.5 μm AL PDs, the bulk leakage current plays a more critical role due to the decreased intrinsic region.

Next, we conducted an optical characterization to investigate the linearity related to epitaxial layer quality after transferring to GMR structures. The optical properties of the fabricated 1 μm AL InGaAs PD on GMR Si with a 100 × 100 μm^2^ optical window, using a 1310 nm laser diode with light coupling through a lensed fiber, showed a clear dependence of photoresponses on incident laser powers (*P*_in_), as shown in Fig. S6g. Without external bias, the InGaAs PD on GMR Si can detect a few hundred-nanowatt input power, where the optical power of laser illumination would be similar to the blackbody radiation^11^. Also, photocurrents (*I*_ph_) as a function of *P*_in_ were plotted from 0 V to verify the linearity of the fabricated PDs with 1 μm AL and 0.5 μm AL, as shown in Fig. 3d. The fabricated InGaAs PD shows linear photoresponses in terms of incident powers for both *T*_AL_. To quantitatively compare the linearity, power-law fitting (*I*_ph_ = *AP*^*α*^) was conducted for the 1310 nm laser, where *I*_ph_, *A*, *P*, and *α* are the photocurrent, a constant for a given wavelength, incident power, and exponent related to linearity, respectively. The definite slopes with 0.94 and 0.98 of *α* for 1 μm and 0.5 μm AL InGaAs PDs on GMR Si were extracted, indicating good photoresponse linearity close to unity, which is a good indicator for fewer junction traps and is very important, especially for imager applications^31^.

On the other hand, the transit time, which is the major parameter for determining the speed of PIN structures, is also an important parameter for high-resolution image sensors because thick AL and scaled pixels can negatively affect electrical and optical crosstalk. From this point of view, the reduction of *T*_AL_ directly leads to the enhancement of transit time reduction for carrier collection and reduced crosstalk. In order to accurately evaluate RF characteristics up to GHz, various considerations need to be changed, including optimization of the pad structure, test patterns for de-embedding, and incident light switching at GHz frequencies. Nevertheless, based on 2-terminal devices, to estimate the speed for the fabricated PDs, we calculated the transit time limited bandwidth and *RC* time limited bandwidth. The detailed calculation and actual *C–V* measurement data are shown in Fig. S6h and the method section. The extraction of 3 dB bandwidth (*f*_3dB_) was plotted in terms of *T*_AL_ for a 15 × 15 μm^2^, which is the smallest one among fabricated devices as depicted in Fig. 3e. While the *f*_3dB_ followed the transit time limited bandwidth with the increase of *T*_AL_, the maximum *f*_3dB_ value was found to be around 0.5 μm *T*_*AL*_, except for parasitic capacitance. It is very helpful to separate the photogenerated carriers, which can result in lowering the electrical crosstalk in image sensors. Also, reducing the *T*_*AL*_ can lead to reduced optical crosstalk which occurs in the back-side illuminated CMOS image sensor (CIS) even without isolation^32,33^. To evaluate the *f*_3dB_ with the same *T*_*AL*_ for various device dimensions, Fig. 3f shows the *f*_3dB_ as a function of device widths. It indicates that the PD with 0.5 μm *T*_*AL*_ can approach the transit time limited region, which corresponds to 60 GHz with a sub 5 × 5 μm^2^ device dimension. When compared to other devices, expected values show superior performances rather than 3.1 GHz core-shell InGaAs PD, and 10 GHz for conventional PIN bulk InGaAs PD^34,35^. Furthermore, the current *f*_3dB_ is determined by considering the intrinsic region of the device without external bias, and the performance can be further improved by applying an additional negative bias to enhance the electric field. From the physical meaning of *f*_3dB_, our devices can inherently achieve transient response speed above 60 GHz. Atually, based on recent progress in high-frame rate imaging, our devices can be utilized in future SWIR high-frame rate image sensors, considering the frame interval is several tens of nanoseconds^16,17^.

### Characterization of spectral responses for PDs with front- and rear-side engineering

**Fig. 4:**
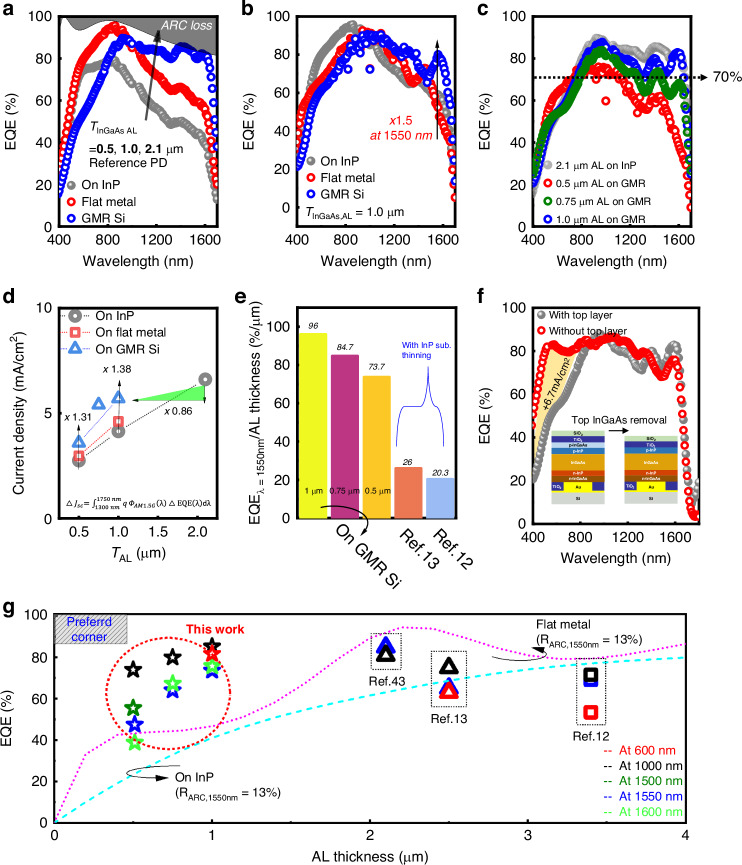
Spectral absorption characteristics of InGaAs PDs. **a** EQE spectra for fabricated PDs on InP substrate with different *T*_AL_. **b** EQE spectra for fabricated 1 μm AL PDs on different bottom structures. **c** Resulting EQE spectra for different *T*_AL_ on GMR structure and reference 2.1 μm AL PDs on InP substrate. **d** Calculated current density using EQE spectrum as a function of *T*_AL_ and structures. **e** Comparison of normalized performances of EQE per *T*_AL_ for proposed devices and conventional PDs. **f** Fabricated devices with/without 20 nm surface InGaAs layer for 1 μm AL PDs on GMR Si. **g** Benchmark for state-of-the-art InGaAs-based SWIR pixels with simulated EQE lines as a function of *T*_AL_ variation (Dashed line: InGaAs PD on InP substrate, dotted line: InGaAs PD on flat metal structure, with the same ARC of this experiment)

By utilizing the advantages of InGaAs materials, we also examined the spectral responses depending on the bottom structures, because the reduction of *T*_*AL*_ should be accompanied by a decrease in the EQE. Figure 4a shows different EQE spectra for reference PDs (on-InP substrate) with different *T*_*AL*_. It is noted that EQE, especially in the longer wavelength region beyond 1000 nm, was dramatically reduced with the decrease of *T*_*AL*_, while half *T*_*AL*_ decreases the EQE by more than 20% around the 1500 nm wavelength.

To systematically evaluate the spectral performances, PDs on GMR Si with different *T*_*AL*_ of 0.5 μm, 0.75 μm, and 1 μm were fabricated. As shown in Fig. 4b, the EQEs as a function of wavelength are measured for the 1 μm AL PDs on different bottom structures. Here, it was found that the EQE enhancement with the FP resonance in the flat metal does not occur beyond 1500 nm, whereas there is a clear improvement in the GMR structure. We clearly identified an increase of EQE of about 1.5 times at 1550 nm by generating additional absorption, which is close to the maximum value limited by DARC. The device with only 1 μm AL showed the peak responsivity of 1 A/W at 1550 nm and detectivity (D*) of 10^13^ Jones which are comparable performances to commercialized detectors as shown in Fig. S7a–c^36^.

Figure 4c is a graph showing the comparison of EQEs relying on the different *T*_AL_ by adopting the GMR structure. It is noteworthy that the structure on 2.1 μm AL on InP and the 1 μm AL PD on GMR Si show almost similar efficiency for the entire spectral region even with such a big difference in the thickness. We observed that the performance of the reference PD can be almost replicated even when the thickness is reduced by half by applying the GMR structure. Additionally, it showed EQE values almost flat with approximately 70% EQE value at the wavelength larger than 800 nm, even in 0.75 μm AL PDs. The 1-µm-thick AL introducing this optical structure to optically excite the GMR for the enhanced broadband EQE could provide the optically comparable EQE of thicker PD (2.1 μm AL on InP), as well as realize a substantial reduction in *T*_AL_ with 2-3 times less. Compared to previous reports, our devices recorded the highest EQEs without gain^37–41^ with the very thin *T*_*AL*_ in the SWIR region considering the major issues for the thickness reduction in the III–V PD^12,13^. Additionally, by integrating the lens or more optimized ARC structures to collect the incoming light significantly on the PD, it might be further improve EQE.

On the other hand, for the 0.5 μm AL PD, as shown in Fig. S7d, it was confirmed that even the short absorption path does not cause the FP resonance in the flat metal structure, while the EQE increases 1.5 times at 1500 nm in the GMR structure. Despite that, the current GMR was fully optimized to 1 µm AL PD, the EQE spectrum for 0.5 μm AL PDs showed slightly degraded EQE near 1550 nm. Due to the shrunk volume of the AL, it is evident that applying the GMR structure of the same period (*P* = 1500 nm) to the thinner PD (*T*_AL_ = 0.5 μm) results in weak optical confinement of the multiple resonances with the high-order diffractions at longer wavelengths. For the thinner AL, therefore, an effective strategy involves reducing the *P* of the gratings further to shift the zeroth-order resonance (*P* < *λ*) to shorter wavelengths, thereby creating a wavelength range for multimode resonances with shorter wavelengths. This strategy allows achieving a broadband EQE exceeding 70% even in a 0.5 μm structure based on the simulated prediction, as shown in Fig. S8. However, the experimentally measured EQE of the 0.5 µm AL with the newly designed GMR may not exceed that of the 0.75 µm AL in the PD when considering the efficient mode confinement in the AL (Fig. S9).

Furthermore, to quantitatively evaluate the improvements, the current densities (*J*) were calculated from 1300 nm to 1750 nm using the equation: $\bigtriangleup J={\int }_{1.3\,\mu {\rm{m}}}^{1.75\,\mu {\rm{m}}}q\varPhi \left(\lambda \right){\rm{EQE}}\left(\lambda \right){\rm{d}}\lambda$ where *λ* is the wavelength, *q* is the charge of the electron, and *Φ*(*λ*) is the photon flux, which in this case was referred to the solar spectrum^42^. As depicted in Fig. 4d, *T*_AL_ scaling decreases the current density over the SWIR region for the reference InP structure. 1 μm AL PDs showed 4.14, 4.61, and 5.7 mA cm^-2^ for on InP, on flat metal, and on GMR Si, respectively, while the 2.1 μm AL PD on InP substrates recorded 6.61 mA cm^-2^. The fabricated PD on GMR Si was dramatically improved of 38% over the reference PD, which is only a 14% decrease compared to the 2.1 μm AL reference PD. Remarkably, only a 19% decrease in current density occurs even in the 0.75 μm AL PD on GMR Si with a 65% reduction in *T*_AL_. For 0.5 μm AL PDs, a 1.31 times higher current density was obtained compared to the 0.5 μm AL reference PD.

To equivalently evaluate the spectral performances with the assumption of the *T*_AL_, we calculated the EQE value at 1550 nm per *T*_AL_ for every reported study^12,13^. As illustrated in Fig. 4e, the previous reports used flip chip bonding and the InP substrate thinning process to enhance visible light absorption, which has the same objective as our PDs. The resulting values revealed 96, 84.7, and 73.7% μm^-1^ for our 1 μm, 0.75 μm, and 0.5 μm AL PDs, respectively. However, conventional PDs showed 26 and 20.3% μm^-1^, respectively. It suggested that the proposed GMR structure can be a very powerful strategy for scaling *T*_*AL*_ for any integration methods, such as flip-chip bonding and monolithic 3D integration. Simultaneously, as proposed, the *T*_AL_ scaling leads to fast carrier collection due to a shorter transit time, which could be highly important for high frame rate and small crosstalk imaging. By using this approach, we verified that the EQE values are still maintained, and photogenerated carriers are rapidly collected.

Furthermore, to maximize visible spectrum absorption, we carried out additional thinning of the p-InGaAs layer in the window region at the photon incident surface. Figure 4f shows the resulting EQE spectra as a function of wavelength depending on the presence of the top p-InGaAs layer with 1 μm AL PDs on GMR Si. With the removal of the top InGaAs layer, the PD showed a remarkably increased EQE exceeding 70% at the visible spectrum, corresponding to 6.7 mA cm^-2^ of current density, while both devices showed similar *I–V* characteristics as shown in Fig. S10a. With only the top InGaAs layer removal, the current density for the visible spectrum in a range from 400 nm to 850 nm was highly improved by 1.17 times, as shown in Fig. S10b. Here, we easily deduced that other *T*_AL_ devices can show the same enhancement of visible absorption as predicted in simulation. So, these results suggest that the proposed thin InGaAs AL PD on the GMR structure and surface layer thinning could lead to highly broadband detection with a high QE in all ranges from 400 nm to 1700 nm. The finding that only 1 μm AL PDs demonstrated ultra-wideband absorption spanning from visible to SWIR spectrum with 80% EQEs is quite noteworthy. This result showed that by using thin AL devices, the proposed devices may effectively answer how to handle the issues with high EQEs and optical/electrical crosstalk. Additionally, the simulation was carried out concerning the scaled pixel, which is equivalent to the latest commercial product with 5 μm as shown in Fig. S11, in order to confirm the applicability to real image sensors in scaled devices^12^. The outcome suggested that scaled pixels could still benefit from the GMR structure.

Finally, Fig. 4g illustrates the benchmark for EQEs as a function of the *T*_AL_ for reported PDs for image sensor pixels (tabluated in Table I in supporting information)^12,13,43^. The dotted lines exhibit the simulation results for expected PD performances at 1550 nm on flat metal and InP bottom structures by utilizing the same epitaxial structures in this work. Compared with conventional pixel designs, in this benchmark, it was observed that the 2 or 3 times decrease in *T*_AL_ was achieved by using the GMR structure with comparable EQE values. Additionally, in the SWIR region, they are unable to surpass the EQEs above the *T*_AL_ limit in a traditional structure. In contrast, the suggested devices successfully overcame the *T*_AL_ limit with a 40% increase at 1550 nm for 1 μm AL PDs. It is convincing that the trade-off between EQEs and *T*_AL_ is successfully solved by our suggested pixel design, suggesting that new device architectures should be developed to meet the demands for high-resolution, broadband, and low-crosstalk image sensors in the future.

In conclusion, our successful demonstration of broadband and highly efficient thin AL InGaAs PDs, utilizing a GMR structure at the rear surface while minimizing visible absorption at the front surface, marks a significant achievement in the field. The introduction of the GMR structure for the SWIR region through optical simulation was implemented by the fabrication of InGaAs PDs with a TiO_x_/Au back surface structure. The proposed thin AL InGaAs PDs resulted in an exceptional ultrawide spectral response, with only a 1 μm AL achieving over 80% EQE across the range of 400 nm to 1800 nm, setting new records in the realm of broadband InGaAs PDs. Moreover, impressive EQE values around 70% were attained throughout the entire spectrum, from visible to SWIR, by utilizing only a 0.75 μm AL PD, which can lead to reduced optical crosstalk. This innovative strategy also promises an improved transit time related to low electrical crosstalk, which is beneficial for high-resolution SWIR image sensors below 5 μm. Additionally, thin AL PDs provide a practical approach for future thin-film broadband imagers, solidifying their potential in the development of advanced SWIR image sensors.

## Materials and methods

### Detailed fabrication process of GMR-integrated InGaAs PDs

The epitaxially grown InGaAs PIN PD structures consisted of p-InGaAs (20 nm)/ p-InP (10 nm)/ i-InGaAs (1000 nm)/ n-InP (5 nm)/ n-InGaAs (20 nm) from top to bottom on a Si substrate with a periodic structure. The metal-organic chemical vapor deposition (MOCVD) epitaxial structures are shown in Fig. S1. This epitaxial structure will be grown on an InP substrate with an inverted structure and flipped onto a Si substrate using wafer bonding. The periodic structure, comprising TiO_x_ and Au with different refractive indices to induce the GMR effect, was simulated with various dimensions by varying the period (*P*) and metal width (*W*), which determine the fill factor (F.F) when the height of 300 nm was fixed (see supplementary materials). These materials were chosen for their suitability for wafer bonding and highly reflective properties in the SWIR region. The 2-inch patterned wafer displayed a fringing pattern indicating interference in patterns, as depicted in Fig. 2b. These patterns, optimized in simulation, consisted of 750 nm width and 1500 nm period. Subsequently, Cr/Au (30/270 nm) metal was deposited by electron-beam (e-beam) evaporation to form the metal region of the GMR structure, with a metal height of 300 nm. A TiO_x_ dielectric layer, with a height of 300 nm, was deposited using an e-beam evaporator, followed by a chemical-mechanical polishing (CMP) process to planarize the rough surface. As shown in Fig. 2c, a flat surface with Au and dielectric widths of 750 nm and a period of 1500 nm was observed in a scanning electron microscopy (SEM) image. This suggested that the wafer-level GMR structure could be well fabricated with a mass-producible fabrication process. Subsequently, the Cr/Au (20/100 nm) metallization process was carried out for an intermediate layer for wafer bonding. The processed wafer was bonded to the Si substrate with a Cr/Au intermediate bonding layer in a wafer bonding chamber for 1 hour at 200 °C, conditions suitable for the monolithic 3D integration process due to low thermal budget. To compare the flat back reflector effects, samples were simultaneously fabricated with only a flat Cr/Au intermediate bonding layer and on-InP reference samples. HCl/H_3_PO_4_-based mixtures were used to fully remove the InP substrate and InP/InGaAs etch stop layers. For fabricating the devices, mesa isolation was carried out with HCl/H_3_PO_4_-based mixtures to expose the bottom InGaAs contact layer for references and bonded sampels. Then, SU-8 passivation layer was adopted to avoide the electrically short from top to bottom eletrodes. SU-8 layer was patterned and hardened by the temperature of 150 ^o^C for 1 h. Finally, Pt/Au top electrode deposition by e-beam evaporation. Then, on-wafer EQE measurement was carried out by set-up as shown in Fig. S12

### Calculation of *f*_3dB_ performances of GMR integrated InGaAs PDs

In order to speculate the 3 dB cut-off limit of the photodetector theoretically, the 3 dB bandwidth (*f*_3dB_) was calculated using these equations: ${f}_{3{\rm{dB}}}=\sqrt{\frac{1}{{{f}_{{\rm{T}}}}^{-2}+{{f}_{{RC}}}^{-2}}}$ ${f}_{{RC}}=\frac{1}{2\pi {RC}}{{f}}_{\,{\rm{T}}}\cong 3.5{v}_{{\rm{sat}}}/2\pi D$ where *f*_*RC*_, *f*_T_, *R*, *v*_sat_, and *D* represent *RC* time limited bandwidth and transit-limited bandwidth, resistance, the saturation drift velocity of carriers in InGaAs, and intrinsic layer thickness, respectively. *R* is assumed to be 50 Ohm of load resistance for calculation. Then, we measured the capacitance (*C*) consisting of parasitic capacitance and junction capacitance by using the LCR meter. To eliminate the parasitic capacitance effect due to the two-terminal and not fully optimized contact structures, we extracted the parasitic capacitance with a small reverse bias of −10 mV by fitting the y-intercept, as shown in Fig. S6h. It shows a parasitic capacitance of 0.94 pF for the 1.0 μm AL PD on GMR Si. Using the two-terminal contact structure of the fabricated devices indicates that our extrinsic structure related to pad schemes was not fully optimized regarding dielectric materials and thickness. By optimizing the extrinsic structures, the parasitic capacitance could be dramatically reduced to 0.1 pF, which is relatively smaller than the junction capacitance^44^. Inset figure shows only the intrinsic capacitance as a function of *T*_AL_ and device area, indicating similar values to the calculated capacitance based on the device area and ideal dielectric constant. We used the ideal junction capacitance for calculating the *RC* time-limited bandwidth.

## Supplementary information

Supporting information

## Data Availability

The data that support the plots within the paper and other findings of this study are available from the corresponding auther upon reasonable request.
