# Supplementary material for: Highly-efficient (>70%) and Wide-spectral (400–1700 nm) sub-micron-thick InGaAs photodiodes for future high-resolution image sensors
Source: Light Sci Appl. 2024 Nov 15;13:311. doi: 10.1038/s41377-024-01652-6 (PMC11568205; doi:10.1038/s41377-024-01652-6)
Supplement: Supplementary file 1 — Supporting information [file 41377_2024_1652_MOESM1_ESM.pdf]

Supplementary Information for

Highly-efficient (>70%) and Wide-spectral (400 nm -1700 nm) sub-micron-thick InGaAs photodiodes for future high resolution image sensors

Dae-Myeong Geum[1,2]+, Jinha Lim[1]+, Junho Jang[1], Seungyeop Ahn[1], SeongKwang Kim[1], Joonsup Shim[1], Bong Ho Kim[1], Juhyuk Park[1], Woo Jin Baek[1], Jaeyong Jeong[1], SangHyeon Kim[1]

[1] School of Electrical Engineering, Korea Advanced Institute of Science and Technology (KAIST), Daejeon 34141, Republic of Korea

[2] Department of Electronic Engineering, Inha University, Incheon, Republic of Korea

Email addresses:

Sang Hyeon Kim (shkim.ee@kaist.ac.kr)

<Reference structure>

Material	Thickness
p^+-InP	200 nm
i-$In_{0.53}Ga_{0.47}As$	2100 nm
n^+-InGaAs	100 nm
Buffer InP	-
InP substrate	

<Thin absorption layer structure>

Material	Thickness
n^+-$In_{0.53}Ga_{0.47}As$	20 nm
n^+-InP	5 nm
i-$In_{0.53}Ga_{0.47}As$	1000, 750 ,500 nm
p^+-InP	10 nm
p^+-$In_{0.53}Ga_{0.47}As$	20 nm
Etch stop InP	10 nm
Etch stop InGaAs	200 nm
InP substrate	

Figure S1. Used epitaxial structures for the fabricated devices

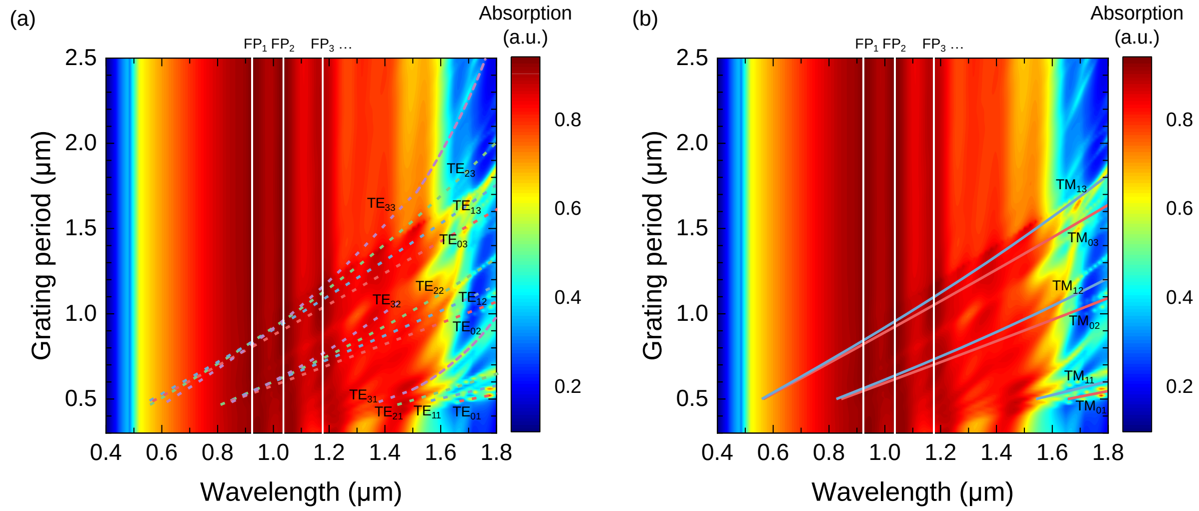

Figure S2. Mode analysis for the (a) TE and (b) TM polarization for the GMR-embedded InGaAs PDs. Dashed (TE modes), and solid lines (TM, and F-P modes) are resonance wavelengths extracted by the analytical model (Contour plots for (a) and (b) are the same with the averaged spectra of the absorption in the TE and TM polarizations.).

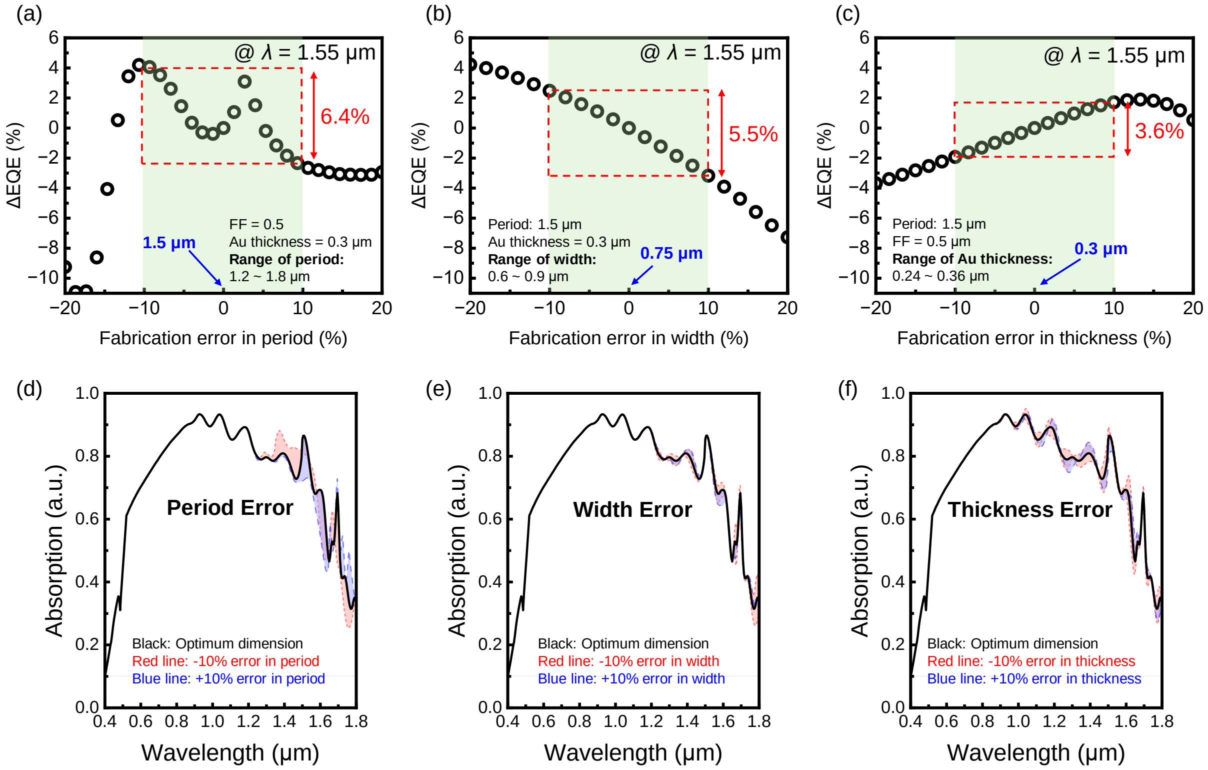

Figure S3. (a)-(c) Variation of EQEs at 1.55 μm considering fabrication tolerances with the period, width, and thickness of the Au. (d)-(e) Simulated absorption spectra of the InGaAs PDs with the dimension ±10% deviated sizes from the optimum dimension.

As shown in Figure S3, the variation in EQEs at 1550 nm wavelength within a ±10% fabrication error for our optimized dimensions is 6.4%, 5.5%, and 3.6% for the period, fill factor, and thickness of the Au grating, respectively. Although our PD was successfully fabricated according to the optimized dimensions of the optical resonant structure, the EQEs are not significantly affected by fabrication tolerances. We also confirmed that the InGaAs PDs with a fabrication error of 10% were quite close to the absorption spectra predicted for the optimum dimensions, considering these tolerances (Fig. S3 (d)-(e)). Among the structural parameters, variations in the spectra due to period errors caused the most significant changes, as the period is crucial in shifting the GMR wavelength. Although a periodically varied GMR

structure enhances a specific band, it can degrade performance in other bands. Therefore, our optimized GMR structure represents the best dimensions to achieve high EQEs over a broadband spectrum. Furthermore, based on this optimization, we successfully fabricated the GMR structure with precise dimensions to enhance broadband EQEs in this work.

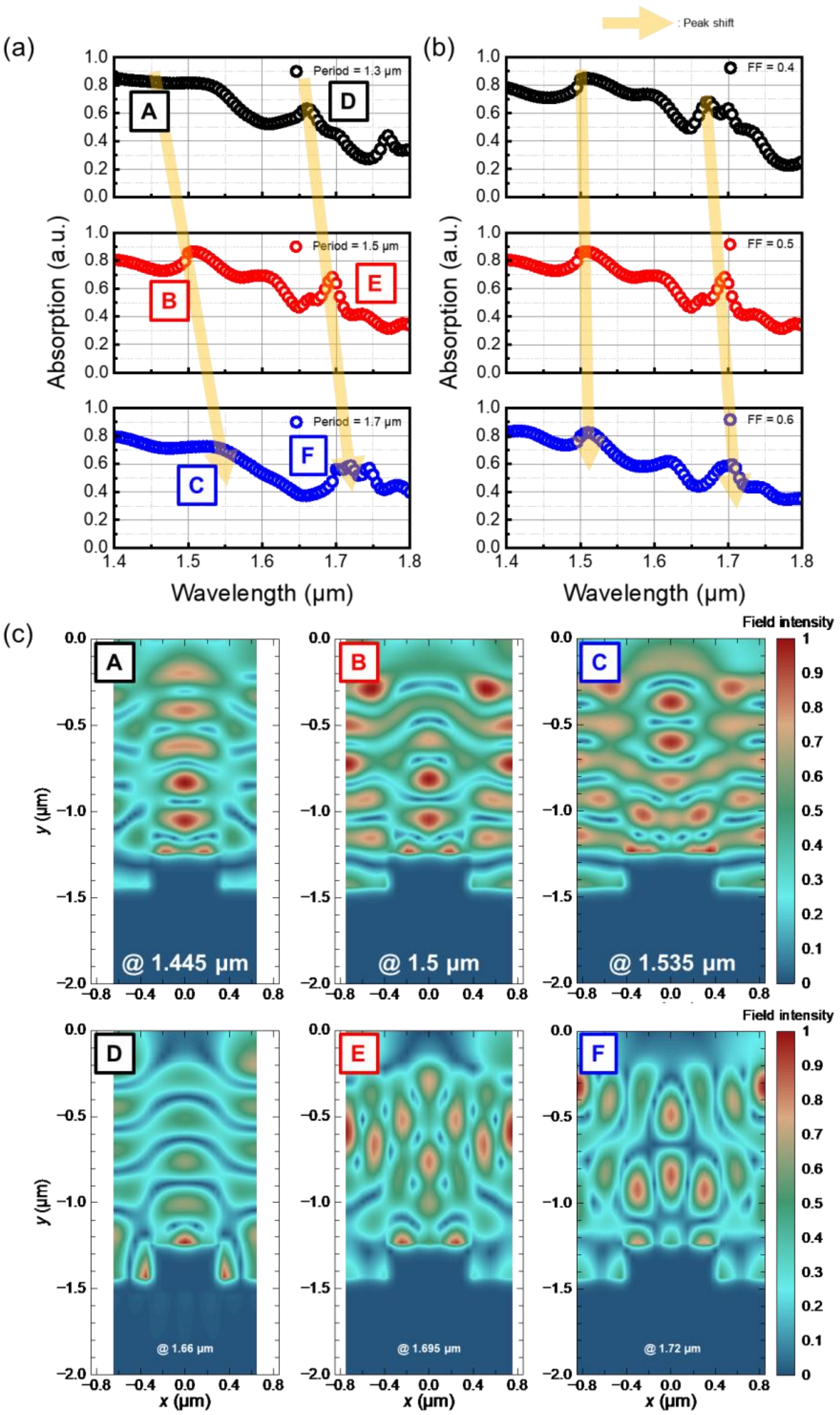

Figure S4. Absorption spectra of the InGaAs PDs as a function of (a) periods (with a fill factor of 0.5) and (b) fill factor (with a period of 1.5 µm). The representative guided-mode resonance mode field distributions corresponding to each resonance in the results of (a).

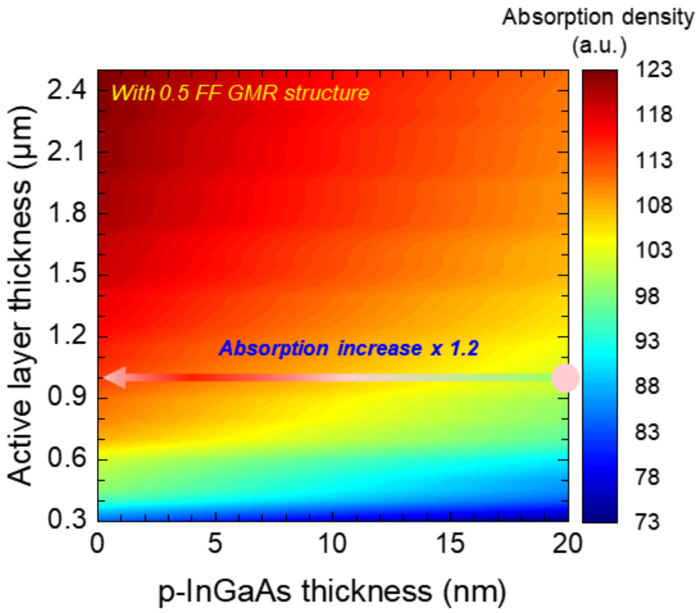

Figure S5. RCWA simulation result for the amount of total absorption from visible to SWIR light depending on the AL thickness and p-InGaAs top surface layer thickness.

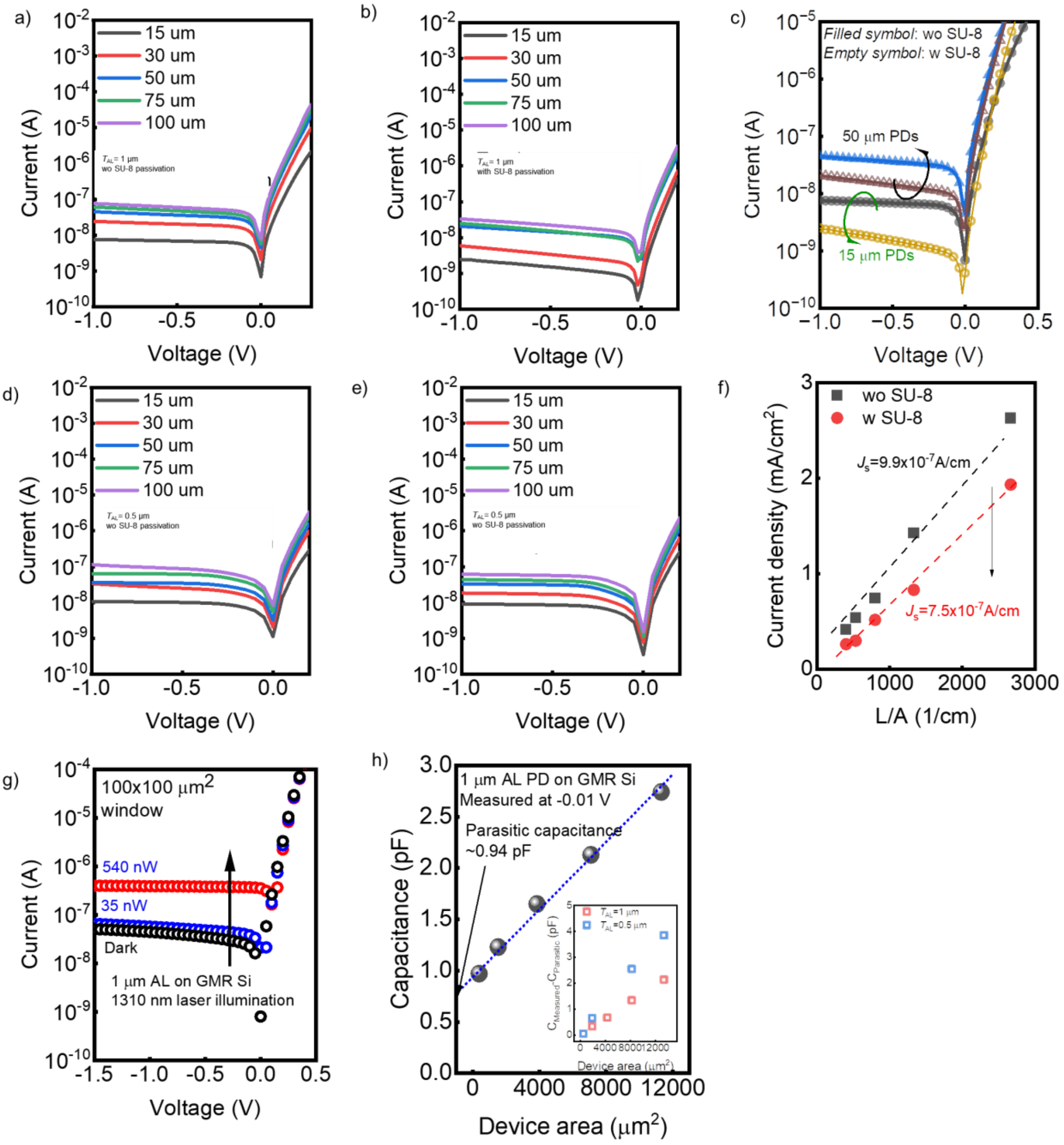

Figure S6. (a) I-V characteristics for the un-passivated 1 μm AL PDs on GMR Si for size-dependent devices (b) I-V characteristics for the passivated 1 μm AL PDs on GMR Si for size-dependent devices (c) Selected I-V characteristics for the comparison for passivation effect (d) I-V characteristics of unpassivated (e) passivated InGaAs PDs with T_{AL}=0.5 um (f) surface leakage current extraction (g) Photoresponses of 1 μm AL PDs on GMR Si with 1310 nm laser illumination (h) C-V measurement data as a function of device area for 1

μm AL PDs on GMR Si and calculated intrinsic capacitance for different T_{AL} as an inset figure.

Fig. S6(a) and 6(b) shows the I-V characteristics for 1 um AL PD on GMR Si with and without SU-8 passivation. The selected devices as shown in Fig. 3(c) demonstrated the reduction of 1 order of magnitude of dark current. When SU-8 was used for surface passivation, It was improved by one order of magnitude indicating SNR would be better. To evaluate the leakage current (dark current) of other devices, we compared the dark current of two samples with significantly different thicknesses of 500 nm and 1000 nm of T_{AL} having same epitaxial structure. It was observed that the dark current shows noticeable degradation even with a T_{AL} of 500 nm, with or without passivation, as shown in Fig. S6(d) and S6(e). Our results showed a slight degradation of dark current in the 500 nm AL, which has a range of 10^{-7} to 10^{-8} A in bias for un-passivated devices. Compared to devices with a 1000 nm active layer, it exhibits a similar current level. This is because all devices are made with a heterojunction structure, providing moderate immunity to surface leakage with the decrease of T_{AL}. In fact, the factor that directly affects surface leakage is more significantly influenced by the fabrication process in this structure. Since all devices were processed identically using wet etching, there is no noticeable difference observed in the *I-V* characteristics.

To quantitatively evaluate the surface leakage current, Fig. S6(f) shows the surface leakage current with and without SU-8 passivation for 0.5 μm AL PDs. Here, it was noted that the passivation effect of SU-8 is less than that of 1.0 μm AL PDs. We reasonably deduced that the decrease of T_{AL} can increase the surface leakage current, but have a relatively small portion of the total current. In fact, as the intrinsic layer thickness decreases, the current would be increased by diffusion current in the p-n junction. So, for 0.5 μm AL PDs, the bulk leakage current would play a more critical role due to the decreased intrinsic region.

Then, we measured the optical properties of the fabricated 1 μm AL InGaAs PD on GMR Si by using a 1310 nm laser diode with a light coupling through the lensed fiber showing clear dependence of photoresponses by incident laser powers (Pin) with a few hundred nanowatt (Fig S6(g), S6(h)).

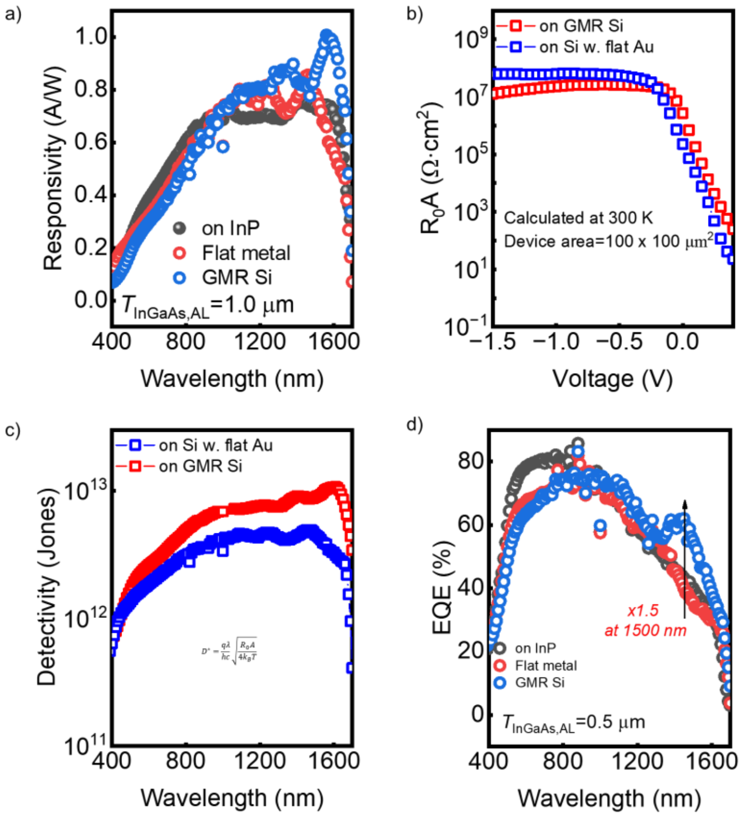

Figure S7. (a) Responsivity as a function of wavelength for different bottom structures with T_{AL}=1um. (b) The extracted R0A values for the devices with GMR Si and flat Au (T_{AL}=1000 nm) and (c) calculated detectivities. (d) Measured EQE spectra for 0.5 μm AL PDs on different bottom structures.

Fig. S7(a) exhibits the responsivity as a function of wavelength for different bottom structures. . When the EQE is converted to responsivity, it shows approximately 1 A/W, which is consistent

with the dark-to-photo current ratios used in commercial devices. Furthermore, we derived the R_0A characteristics of the device which infer the noise characteristics of our devices in detectivity (D*) value. As shown in Fig S7(b), and S7(c), it was confirmed that there is a clear enhancement of D* value in GMR Si devices compared to flat Au structure. With similar R_0A values in both devices, the GMR Si structure can obtain a higher EQE due to the guided mode structure. The D* value with GMR Si devices revealed comparable performances in the full spectral range to commercially available InGaAs diode structure at 300 K Fig. S7(d) shows the measured EQE spectra for 0.5 um AL PD on GMR Si depending on the different bottom structures. Unlike 0.75 um AL PD on GMR Si, Fabry-Perot resonance does not occur in flat metal Si structure due to insufficient AL thickness. In contrast, it can be clearly seen that there is a 20% improvement at 1500 nm for GMR Si structure. From this result, it was concluded that the GMR effect can effectively enhance the EQE at longer wavelength region even with very thin AL layer.

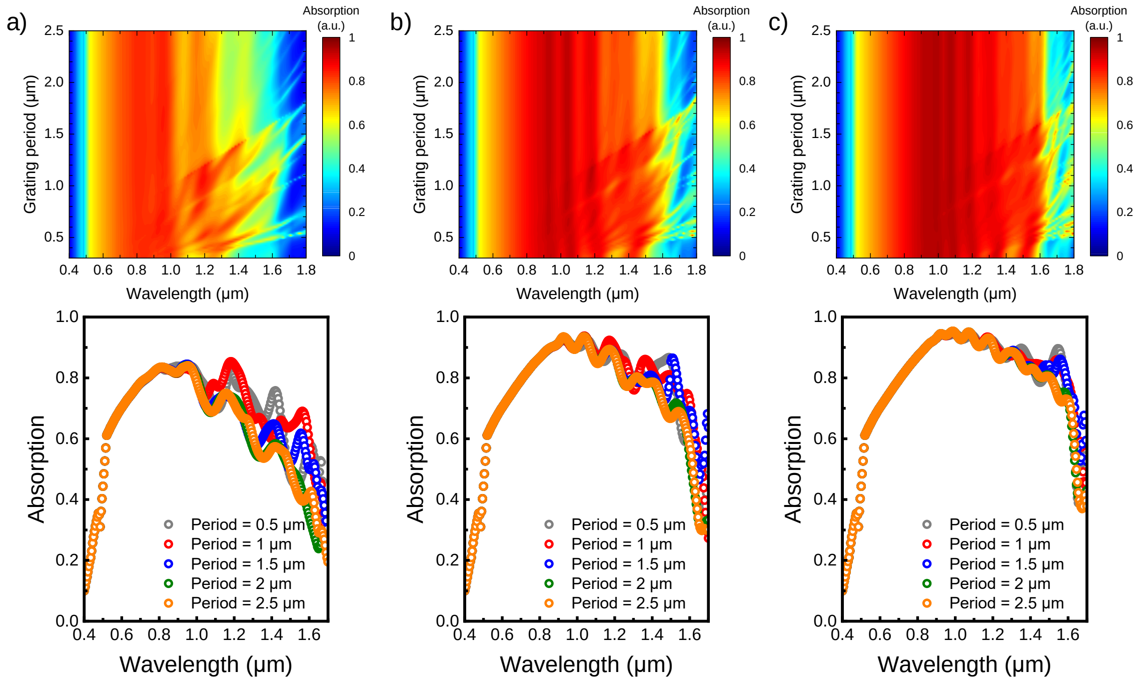

Figure S8. 2D mapping simulation result for the total amount of absorption regarding various AL thickness on various GMR structure with different periods and selected absorption spectra as a function of wavelength for (a) 0.5 µm AL PDs on GMR (b) 1.0 µm AL PDs on GMR (c) 1.5 µm AL PDs on GMR.

Fig. S8 shows RCWA simulation results for absorption behaviors depending on different AL thickness and periodic structure periods. As shown in Fig. S8(a), the current GMR period could not be effectively enhance EQE at the longer wavelength region. Thinner AL PD required the shorter period of GMR due to enhance the confinement of multimode absorption. Fig. S8(b) and S8(c) exhibited the 1 um AL and 1.5 um AL PD, indicating there are clear performance improvements near the band edge.

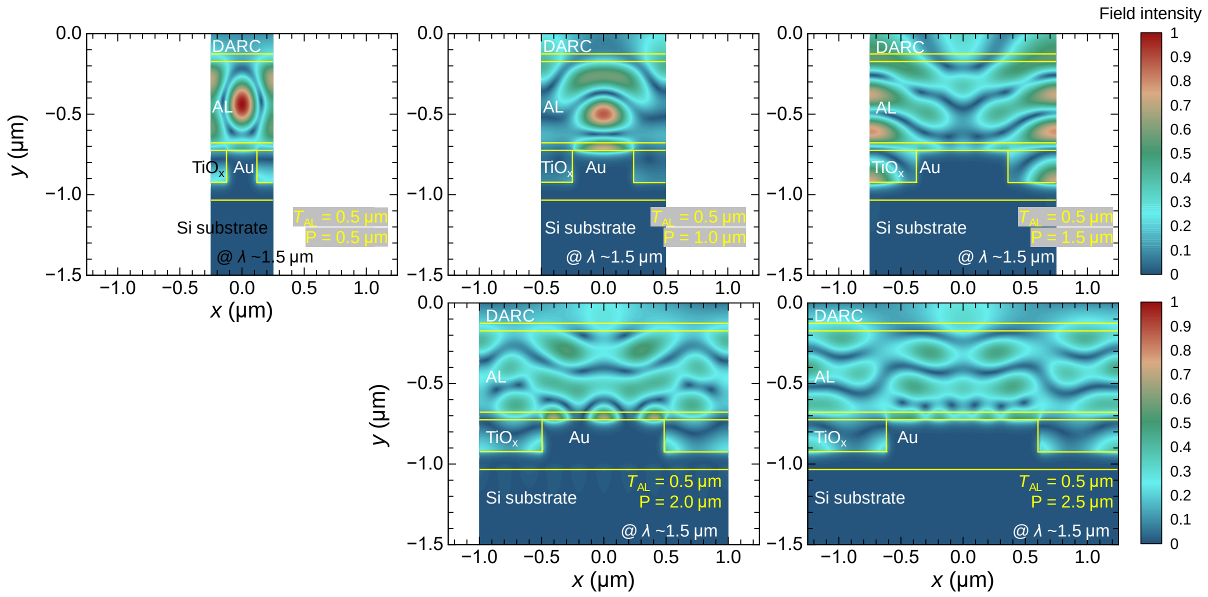

Figure S9. (a) Normalized field distribution of 0.5 μm AL InGaAs PIN PDs by varying the period of the GMR structure (P = 0.5 ~ 2.5 μm) at each resonant peak around 1.5 μm wavelength.

Figure S6 illustrates the field distributions for 0.5-μm-thick AL InGaAs PDs on GMR structures, showcasing varying grating periods (P = 0.5, 1.0, 1.5, 2.0, and 2.5 μm) at resonant peaks around a 1.5 μm wavelength (Fig. S5(a)). The field intensities were normalized based on the maximum value observed when P = 0.5 μm, which exhibited the highest intensity. Beyond the period of 1.5 μm in the GMRs, weaker field confinement of optical resonance with high-order diffraction occurred, while stronger fields associated with zeroth modes were localized in the ALs for P values of 0.5 and 1.0 μm. Considering those high-order mode resonances in ALs thinner than 1 μm result in weak absorption, tuning the period of the GMR to induce a spectral shift for zeroth-mode resonance at longer wavelengths in the SWIR may be the most efficient approach.

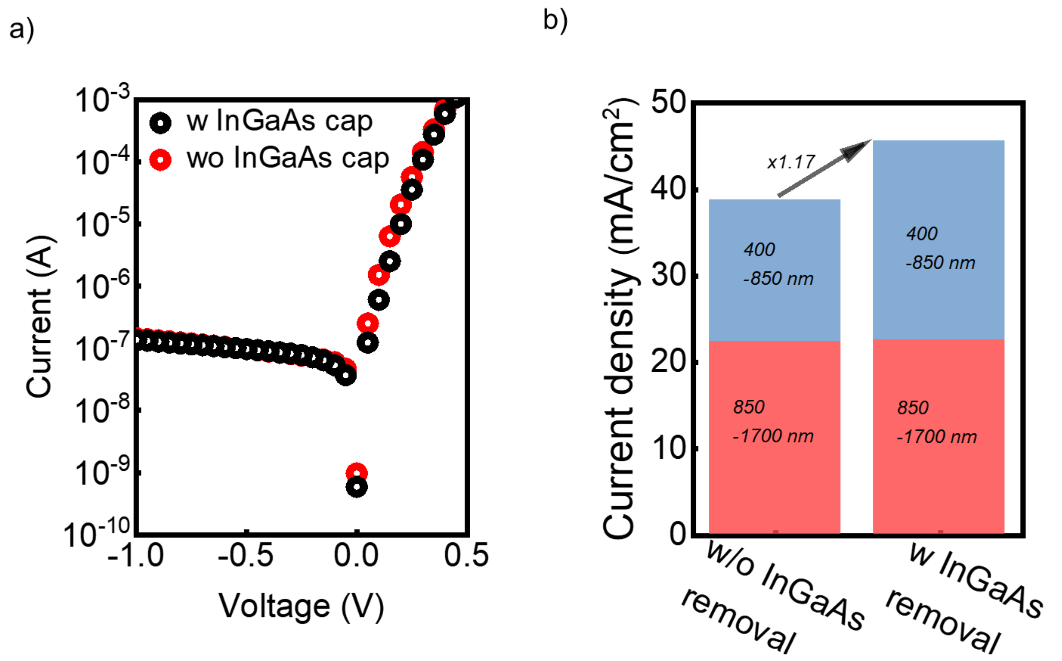

Figure S10. (a) I-V characteristics with and without p-InGaAs surface layer removal (b) Calculated current densities depending on the visible and IR absorption region with and without surface layer elimination.

To maximize the visible spectrum absorption, we carried out the additional thinning of the p-InGaAs layer in the window region. With p-InGaAs removal process, I-V characteristic shows the negligible change and provides remarkably increased QE at visible spectrum corresponding to 6.7 mA/cm^2 of current density as shown in Fig. S7(a) and S7(b). These results suggested that the proposed thin InGaAs AL PD on the GMR structure and contact layer thinning could lead to highly broadband detection with a high QE in all ranges from 400 nm to 1700 nm.

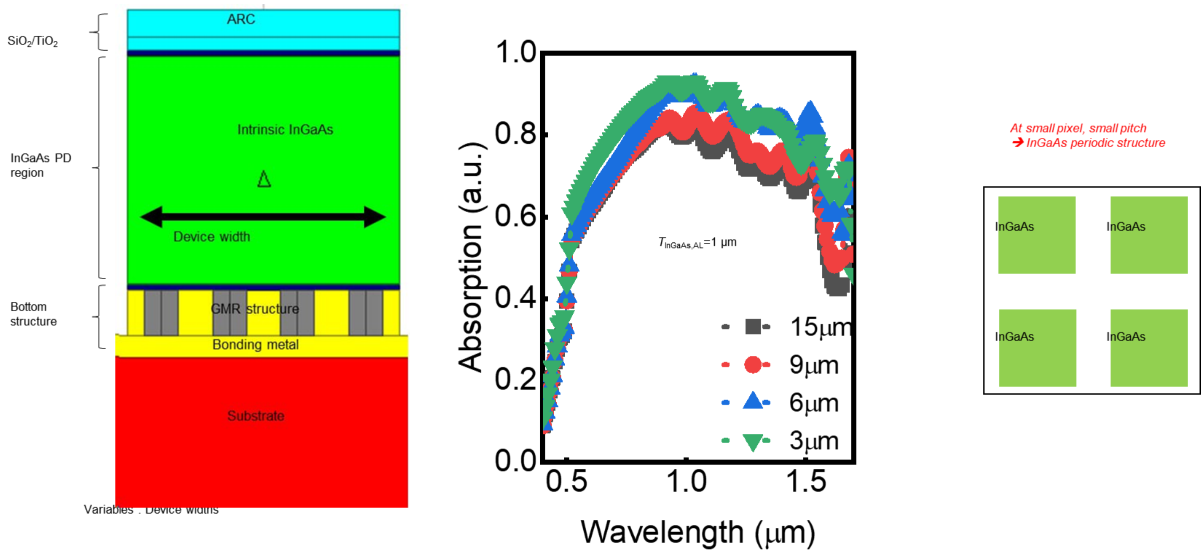

Figure S11. RCWA simulation structure and design parameters for scaled pixel simulation and their simulated absorption spectra depending on the different pixel sizes with 1 μm AL PDs on designed GMR Si.

To evaluate the effectiveness of GMR integrated structures, we carried out the simulation of scaled pixel condition with same period and epitaxial structures. For the 1 um AL PD on GMR Si, the simulated structure and the result were shown in Fig. S8. As a result, this GMR structure was not only for a very large film detector but also applicable to the scaled pixel for image sensor (comparable size to the recent commercial product, 5 μm).

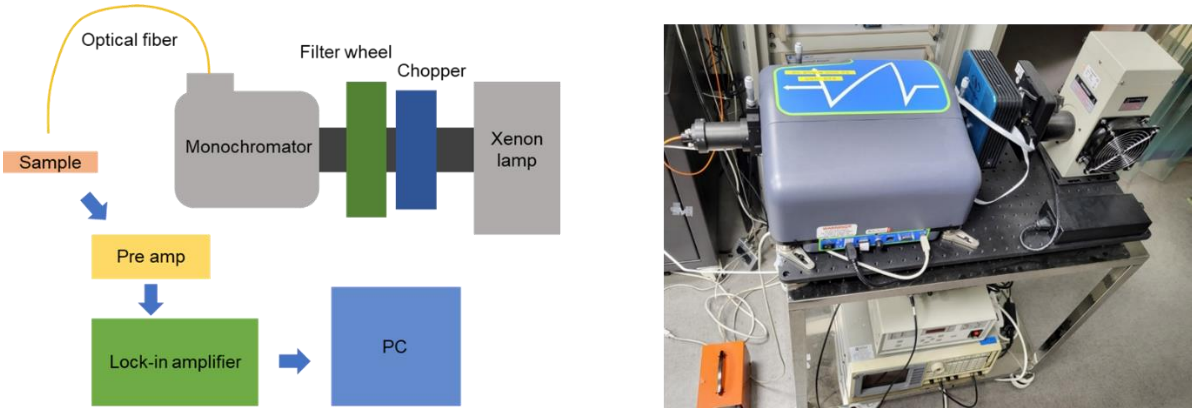

Figure S12. On-wafer EQE measurement set-up schematic and picture.

White light from a Xenon lamp is split into a monochromatic light by a monochromator and transferred through an optical fiber.

Table.I Benchmark about InGaAs photodiodes which ever reported

References	Structure	T_{AL} (nm)	J_{dark}	EQE (%) @1550 nm	EQE/T_{AL} (%/μm) @ 1550 nm	Estimated f_{3dB} (GHz) (from Fig.3e)
**This work**	Mesa type	500	~10^{-7} A/cm^2 @ -1V for 50 μm devices	47.5	95	44.8
		750		64	85.3	36.8
		1000		73.7	73.7	28.9
Ref. 12		2100	~10^{-7} A/cm^2 @-1V for 40 μm devices	81	38.6	14.7
Ref. 13	Planar type	2500	-	65	26	11.8
Ref. 36		3400	-	69	22.3	8.6
